# Patent and Marketing Exclusivities 101 for Drug Developers

**DOI:** 10.2174/1872208317666230111105223

**Published:** 2023-04-05

**Authors:** Bryan Oronsky, Scott Caroen, Franck Brinkhaus, Tony Reid, Meaghan Stirn, Raj Kumar

**Affiliations:** 1 EpicentRx, Torrey Pines, San Diego, California, USA

**Keywords:** Patents, regulatory exclusivities, RRx-001, NLRP3 inhibitor, radioprotection, anticancer

## Abstract

Despite an ever-increasing need for newer, safer, more effective, and more affordable therapies to treat a multitude of diseases and conditions, drug development takes too long, costs too much, and is too uncertain to be undertaken without the conferment of exclusionary rights or entry barriers to motivate and sustain investment in it. These entry barriers take the form of patents that protect intellectual property and marketing exclusivity provisions that are provided by statute. This review focuses on the basic ins and outs of regulatory and patent exclusivities for which new chemical entities (NCEs), referring to never-before approved drugs with an entirely new active ingredient, are eligible and uses RRx-001, a small molecule aerospace-derived NCE in development for the treatment of cancer, radiation toxicity, and diseases of the NLR family pyrin domain containing 3 (NLRP3) inflammasome, as a “real world” example. This is intended as a ‘101-type’ of primer; its aim is to help developers of original pharmaceuticals navigate the maze of patents, other IP regulations, and statutory exclusivities in major markets so that they can make proper use of them.

## 1 INTRODUCTION

The year 2021 marked the 50^th^ anniversary of the National Cancer Act of 1971, signed into law by Former President Richard Nixon, which launched what has been euphemistically referred to as the “war on cancer” [1]. Since then several new wars have been opened on the therapeutic fronts of obesity, diabetes, Alzheimer’s, heart disease, human immunodeficiency virus (HIV), coronavirus disease of 2019 (COVID-19), *etc*. and paramount to their continuation (and hopefully eventual success) are the grant of patents and market exclusivities on which pharmaceutical companies are dependent to recoup the enormous attendant research, development, regulatory, and post-marketing costs.

It is estimated that for every one drug to reach clinical approval at an average cost of $1-2 billion and a duration of 10-15 years [2], nine preceding drugs have failed. Such a high failure rate discourages real innovation in favor of “pseudo-innovation” with substitute “me too” drugs, trivial formulation changes, and drug combinations that are potentially no better, or only incrementally better, than their predecessors but that are ‘safer bets’, having been ‘de-risked’ in terms of cost, duration, and the likelihood of approvals. This process of incremental patenting, which is colloquially known as “evergreening” because it attempts to extend or “evergreen” the exclusivity period of a drug effectively constitutes the path of least resistance, being much cheaper and easier than radical innovation [3].

Regardless of the specific figures, because historically, fewer than one in ten drug candidates succeed, and because the patent system itself is very costly in terms of attorney’s fees and litigation, the profit margins from the few drugs which are approved must exceed global development costs, including the costs of failures that have occurred along the way [4, 5] This also explains the relative focus on therapeutic areas with larger patient pools and, hence, more economic potential, like oncology, immunology, endocrinology, neurology, and cardiology where the ability to recoup the R&D investment is higher [6].

The result is that for some diseases, particularly rare diseases, few, if any, treatment options exist, while for others a surplus of similar, overlapping medicines are available, all competing for a share of an established, lucrative market [7]. The obvious benefit from the competition is lower prices and greater access to pharmaceuticals for patients [8]. On the flip side, however, potentially to the detriment of patients, innovation tends to decline with competition, as competition reduces profitability.

To remedy the latter, patent law and drug regulations reward innovation in the short run with a *de facto* monopoly for a predefined, set period, during which time competition is effectively stifled and supra-competitive monopoly prices can be charged, so that the costs of innovation are fully compensated, thus incentivizing follow-on innovation [9]. Then, once the window of exclusivity ends and generics (*i.e*., bioequivalent versions of the branded drug) enter, competition forces prices back down to earth as competitors are all vying for a piece of the pie [10, 11]. A case in point is Eli Lilly’s blockbuster antidepressant, Prozac^®^, whose patent expiration resulted in a loss of almost 70 percent of its market share within 20 weeks of generic entry [12].

This delicate balancing act between the “carrot” of incentivization to innovate, which may temporarily lead to higher-than-competitive prices and inequitable access to medicines, *versus* the “stick” of competition to reduce prices for increased patient access once the monopoly expires is central to statutory and patent policies [13]. In the absence of these incentives, no biopharmaceutical company would likely be motivated to develop new treatments otherwise known as new chemical entities (NCEs), new molecular entities (NMEs), or new biological entities (NBEs), given the serious financial commitment involved; free-riding competitors at much less expense could and would manufacture and market reverse engineered (and possibly even improved) versions of the same treatments, undercutting the price, and denying the original innovating company a profit or even the chance to break even.

This review attempts to demonstrate regulatory and patent exclusions “in action” as they apply to a *bona fide*, first-in-class NCE like RRx-001. It focuses on the major pharmaceutical markets of the “Big Three”, the United States, the European Union (EU), and Japan. Exclusivities for biologics, which are more complicated and costly to develop than NCEs, are not covered in this case study and will be the subject of another review.

## 2 RRx-001

This small molecule NCE, currently in a Phase 3 trial for the treatment of small cell lung cancer [14, 15], an orphan indication, and a soon-to-start Phase 2/3 trial in first-line head and neck cancer for the protection against oral mucositis arose from a collaboration between the biopharmaceutical company, EpicentRx (formerly RadioRx) and ATK (now Northrop Grumman), an aerospace and defense contractor. RRx-001 is a highly energetic material due to the presence of a strained four-membered ring, called a dinitroazetidine, which decomposes cytotoxically under hypoxic conditions that are endemic to tumors. Its closest chemical congener is a component of rocket fuel, 1,3,3 trinitroazetidine (TNAZ) [16, 17].

This is the first time in the annals of medicine that a dinitroazetidine, which is explosive to manufacture, and requires the use of specialized energetic production facilities with highly trained personnel, has ever been evaluated for therapeutic purposes. To render the final active pharmaceutical ingredient (API) non-explosive for safe storage and transport involves a proprietary manufacturing process. In recognition of the *sui generis* and first-in-class chemical structure and molecular mode of action of RRx-001, United States Adopted Names (USAN) and International Nonproprietary Names (USAN/INN) have assigned a standalone name that could be a future new stem.

The indications for which RRx-001 is under study are varied and include cancer, [18] autoimmune [19, 20], metabolic (Morgensztern *et al*. 2019), and inflammatory diseases [21, 22] (Chen Y *et al*. 2021), neurodegenerative diseases, infections, [23] radioprotection, [24] space exploration [25], and disorders characterized by ischemia-reperfusion injuries such as myocardial infarction and stroke [26]. To mitigate the main toxicities of the drug, pain, and venous inflammation and thrombosis on infusion, observed in 12 clinical trials and 300+ patients, requires the use of an *ex vivo* device in which a sample of the patient’s blood is mixed with RRx-001 prior to intravenous administration; hence RRx-001 has been designated as a drug-device combination. These characteristics and properties of the drug as they relate to patent and regulatory exclusivities will be explored in more detail below.

## 3 PATENTS

A patent is a legally enforceable grant from a government to the patentee for a set period (typically 20 years), which may be substantially less in practice, with rights to exclude others from making, using, selling, offering to sell, and importing the patented invention in exchange for a comprehensive disclosure of the invention [27]. This is the time-honored quid pro quo or trade-off of a patent: exclusivity for a limited period contingent on the description of an invention, which meets the patentability requirements of novelty, utility, and non-obviousness, in sufficient detail such that one of ordinary skill in the art may practice it [28]. The legal right of exclusion or *ius prohibendi* is as broad or as narrow in scope as the patent’s claims, which define its “metes and bounds” [29]; like a picket fence that demarcates the limits of privately owned parcels of land to prevent trespass, the claims cover in words the boundaries of the intellectual property and, like an actual trespass, patent infringement is a tort [30].

By statute, claims take two forms: independent claims and dependent claims. Independent claims stand alone and define the invention without reference to any other claims; dependent claims refer to one or more previous claims and may more narrowly define the invention [31]. A claim to a whole class of entities that share a common property is often referred to as a “genus” or generic claim, whereas claims that cover only a single entity are often called “species” claims.

Since 1995, the Trade-Related Aspects of Intellectual Property Rights (TRIPS) harmonization agreement requires all members of the World Trade Organization [32] (today more than 150 countries) to adhere to minimum standards for intellectual property governance, including patents on medicines. These standards include a 20-year term of protection for pharmaceutical products and processes against “unfair commercial use”, to which WTO members must conform on penalty of losing trade advantages [32]. In 2013, in line with the rest of the world, the United States switched from a first-to-invent to a first-to-file system, whereby the first individual(s) to file is typically entitled to the patent, even if another individual(s) invented it first [33].

TRIPS harmonization aside, patent protection remains highly balkanized, involving different countries and patent offices. Moreover, the exclusive rights granted by a patent in one territory or country are geographically constrained to that territory or country and do not extend outside of it [34]. In this way, for example, US-registered patents are only valid in the United States, not in Europe, Japan, or Korea. To protect the same invention in Europe, Japan or Korea requires that a separate patent application is filed at the European Patent Office (EPO) or the various European national patent offices, the Japan Patent Office (JPO), or the Korean Intellectual Property Office (KIPO), either directly or *via* the Patent Cooperation Treaty (PCT) administered by the World Intellectual Property Organization [35] in up to 193 countries. (https://www.wipo.int/pct/en/pct_contracting_states.html) Moreover, according to the principle of “patent independence”, the adjudication of a patent in one country is, in theory, if not always in practice, separate from its adjudication in another so that the decision to grant or reject a patent application is made on a country-by-country basis and not simply ‘rubber-stamped’ between patent offices [36, 37].

### 3.1 Patent Categories

Categories of pharmaceutical patents include (i) composition of matter or product, which claim the active ingredient(s) in a drug as a previously unknown new chemical entity; (ii) process, which covers a particular process used to make or manufacture the drug; (iii) method-of-use or indication, which cover the medical indication; and (iv) formulation that cover both the active pharmaceutical ingredients of a drug and the non-active carriers or excipients, such as fillers, binders, disintegrants and lubricants that constitute the final dosage form, which is administered to the patient as tablets, capsules, injectable formulations, transdermal patches, *etc*. (v) drug-device, which cover the drug and a device, which is integral to its administration (vi) crystal, which cover crystalline structures such as polymorphs, salts, solvates, hydrates, and pharmaceutical cocrystals.

From the standpoint of the patentee, the composition of the matter patent is the most desirable, since it affords the broadest protection, which extends to all uses and all forms of the drug, regardless of whether those uses or forms were disclosed at the time of filing. Nevertheless, the average effective composition of matter patent term on a new drug is effectively 8-12 years, given that the clock starts ticking at filing and the clinical development times are long [38].

So-called “tertiary” drug-device combination and crystal patents are also highly attractive in the United States (US), the former because the drug and the device, qualify for inclusion in the United States Federal Drug Administration (USFDA’s) “Orange Book” as a combination product, are separately patentable; this means that if the patents on the device component expire later than the patents on the medicine component, generic entry may still be prevented; and the latter because crystal forms, *e.g*., polymorphs, hydrates, solvates, and co-crystals are not only themselves patentable but they may also be eligible for Abbreviated New Drug Application (ANDA) approval since the FDA defines them as “different crystalline forms of the same API” [39].

So-called “secondary” patents, which cover process, indication (method of use), and formulation, may offer comparatively less protection than more valuable “primary” or core composition of matter patents. On the other hand, the use of patents in the Orange Book that span all the approved indications may provide a significant fence of exclusivity around the product. A weakness can arise where a product is approved for multiple indications, composition coverage is expired, and there is only patent protection for one or a subset of the approved indications. In that case, a generic can seek approval based solely on the approved indication that is not protected by a patent and thereby try to design around the existing patents.

### 3.2 Patent Term Extensions (PTEs) and Supplementary Protection Certificates (SPCs) of up to 5 years and Patent Term Adjustments (PTAs)

In over 60 countries, NCEs are eligible for Patent Term Extensions (PTEs) as they are called in the United States, [40] or Supplementary Protection Certificates (SPCs) as they are called in Europe. Depending on the country, such extensions may have a duration of up to 5 years. This is in compensation for the lengthy clinical development and new drug application/marketing authorization periods, during which time it is not possible to market the invention. In the United States, the remaining patent life after PTE may not extend beyond 14 years after approval, and similar restrictions may apply in other countries. Furthermore, although there are variations among different countries, a PTE/SPC typically only applies to the first approval of a new active ingredient, and typically only one patent may be extended per new active ingredient.

A PTA, whose term varies, is used to compensate for administrative delays during patent prosecution and is available in the United States and certain other countries [41a].

## 4 PATENT EXCLUSIVITY “IN ACTION” WITH RRx-001

To date, over 50 patent applications have been filed relating to RRx-001 and numerous patents have been granted.

### 4.1 First Composition of Matter Patent

International Patent Application No. PCT/US2006/031917 [41b] and corresponding United States Patent Application No. 11/502,810, which were filed in August 2006, describe the compound RRx-001 (also known by its chemical acronym, ABDNAZ (alpha bromodinitroazetidine)), a synthetic method for RRx-001, and therapeutic uses in cancer, autoimmune, and inflammatory diseases. Multiple patents have been issued in the United States and foreign countries stemming from these applications, which, depending on the country, have claims covering the compound and pharmaceutical compositions, uses, and synthesis thereof.

In the US, one patent from this family will have a projected term to 2031, factoring in PTE, with the remaining patents having a term to 2026. Similarly, the patent in Europe has a projected term to 2031 factoring in SPC. According to the preparation method described in this patent family, the final crystalline API is variably sensitive to several stimuli, including impact (or shock), friction, and electrostatic discharge, as defined by specialist tests. The cause of the variably sensitive behavior is complex, with no theoretical, a priori way to predict it. This potentially poses a problem for handling, shipping, and storage. Fortunately, and for an unknown reason, despite an extensive investigation by chemists experienced in explosive methods, the batch of RRx-001 that was manufactured for Phase 1 and 2 clinical trials is insensitive to external stimuli.

### 4.2 Second Composition of Matter Patent

An International Patent Application No. PCT/US2019/032780 [41c], which was filed in June 2022, and has a projected term, if and where granted, to approximately 2042, covers a new synthetic method and resultant form of the compound that renders the final crystalline API consistently (instead of variably from the original synthetic method) non-explosive with improved solubility and therapeutic activity. Non-explosivity is, of course, important for safe storage, handling, and transport. However, such is the shock- and heat-sensitivity of the intermediates of RRx-001, which are on par with several benchmark explosives such as octahydro-1,3,5,7-tetranitro-1,3,5,7-tetrazocine (HMX) and 1,3,5-trinitroperhydro-1,3,5-triazine (RDX), that only a very few specialized facilities with an intimate understanding of not only Current Good Manufacturing Practice regulations (cGMP) but also explosives and the precise technique which is required to handle them are qualified to manufacture RRx-001.

### 4.3 Drug and Device Patents

International Patent Application No. PCT/US2019/036588 [41d], which was filed in June 2019, and has a projected term, if and where granted, to approximately 2039, covers the use of external devices for the administration of RRx-001. The FDA has designated RRx-001 as a drug-device combination since an external device is needed to deliver it safely *via* IV. This designation is highly advantageous especially in the United States given that a putative abbreviated new drug application (ANDA) filer must agree to only enter the market when each patent listed in the FDA Orange Book either expires (paragraph III certification), or is invalid, unenforceable, or will not be infringed by the product (paragraph IV certification). If the putative ANDA filer makes a paragraph IV certification, the new drug application (NDA) holder is entitled to sue for patent infringement before the proposed generic product is made, used, or sold. In addition, paragraph IV suits trigger an automatic 30-month stay of FDA approval of the generic product application.

### 4.4 Co-crystal Patents

A provisional patent was filed in March 2022 with a projected term, if and where granted, to approximately 2043, that covers co-crystals of RRx-001 with improved physiochemical properties. These are crystalline solid forms that incorporate two or more components in the same crystal lattice, one being the active pharmaceutical ingredient (API) molecule and the other a cocrystal former or coformer. These coformers are generally recognized as safe (GRAS) [42]. The importance of this patent is that, as stated above, co-crystal patents can extend patent rights for the project and may also meet regulatory requirements for bioequivalence; this means that it may be possible to file for approval under the much simpler ANDA route.

### 4.5 Method of Use/Indication Patents

Multiple patents have been filed for RRx-001 use in cancer, and in autoimmune, inflammatory, neurodegenerative, and infectious indications.

## 5 REGULATORY EXCLUSIVITIES

**Fig. (1):**
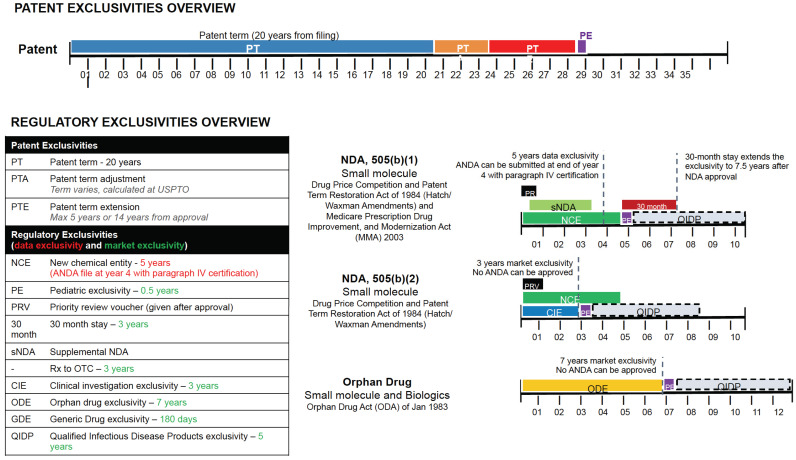
Overview of patent and regulatory exclusivities in the US. Adapted and used under Creative Commons CC-BY license from Peng, B., & Tomas, M. C. (2014). A cheat sheet to navigate the complex maze of exclusivities in the United States. Pharmaceutical patent analyst, 3(4), 339–343. https://doi.org/10.4155/ppa.14.30. PMID: 25291306.

In contrast to the one-size-fits-all patent system, regulatory exclusivity periods, which may run concurrently with or extend beyond the terms of patent protection, are designed to defer the entry of generic competitors. These regulatory exclusivities, also known as “pseudo-patents”, [43] because they confer a limited patent-like monopoly on the holder, specifically encourage certain forms of research and development (R&D), such as the development of orphan drugs or new chemical entities or the deployment of approved drugs for new uses or for use in children (Fig. **1**). Data exclusivity, a term that originated in the US, refers to a statutory provision wherein the innovator’s preclinical and clinical data or information remain “off limits” to would-be generic competitors, which prevents these competitors during that time from filing a new drug application/marketing authorization based on the innovator’s preclinical or clinical data [44, 45]. In contrast, market exclusivity refers to a period during which an exclusive right to market the product is granted.

## 6 EXCLUSIVITY IN THE US

In 1984, to level the playing field, the Drug Competition and Patent Term Restoration Act (Hatch-Waxman) introduced the ‘Abbreviated New Drug Application’ (ANDA) for generic drugs, which supports previous safety and efficacy data for the innovator drug, which only requires bioequivalence studies *in lieu* of more expensive clinical trials [46]. If the generic company obtains the right to cross-reference the original data, then it may submit a supplemental NDA or ANDA, abbreviated as supplemental new drug administration (sNDA) or supplemental abbreviated new drug administration (sANDA) for any changes in labeling, formulation, patient population, manufacturing, *etc*. [47, 48].

### 6.1 5 Year New Chemical Entity Exclusivity

To compensate the innovator for the loss of market share, the Act introduced a period of 5 years of data exclusivity, which only applies to an NCE like RRx-001 [49]. In practice, this period may be extended to up to 7.5 years since it is common for the brand name company to file a patent infringement lawsuit against a generic applicant, once it receives notice of an ANDA submission, triggering a 30-month stay, during which time the FDA cannot approve the ANDA so long as the infringement lawsuit is ongoing or resolved in the brand name company’s favor [50].

### 6.2 5 Year New Antibiotic Exclusivity

To combat microbial resistance and to incentivize the development of new antibiotics, the US Congress enacted the Generating Antibiotic Incentives Now Act (GAIN Act) of 2012, which adds 5

years of additional nonpatent exclusivity to manufacturers of Qualified Infectious Disease Products (QIDPs) [51]. A QIDP is defined as “an antibacterial or antifungal drug for human use intended to treat serious or life-threatening infections, including those caused by - (1) an antibacterial or antifungal resistant pathogen, including novel or emerging infectious pathogens; or (2) qualifying pathogens listed by the Secretary under subsection (f) [of section 505E of the Food, Drug, and Cosmetic Act (FD&C Act)].”

### 6.3 3 Year New Clinical Investigation Exclusivity

In addition to 5 years of data exclusivity for all NCEs, the Hatch-Waxman Act also includes a three-year period of exclusivity for a previously approved drug product that contains an active ingredient, should another NDA for that drug be approved for a new use, a new formulation, a different strength, dosage form, or route of administration. As an example, RRx-001 is expected to be evaluated for several different indications: 1) small cell lung cancer (SCLC) 2) mucositis 3) radiation countermeasure 4) leukemia and myelodysplastic syndrome 5) NLRP3 inflammasome-related diseases.

### 6.4 Orphan Drug Exclusivity

This provides 7-years of exclusivity for drugs, known as “orphan drugs”, that treat or prevents rare but serious diseases. The 7 years of exclusivity begins on the date of NDA approval for the orphan indication [52]. To date, RRx-001 has been granted orphan designation in SCLC, acute radiation syndrome, neuroendocrine disease, and glioblastoma.

### 6.5 Pediatric Exclusivity

This provides 6 months of additional exclusivity to other patent and regulatory exclusivities, provided at least 9 months of patent term or data exclusivity remain, on which “pediatric exclusivity” can attach if pediatric studies are performed [53]. RRx-001 has started a trial called PIRATE (ClinicalTrials.gov Identifier: NCT04525014) in children with recurrent or progressive malignant solid and central nervous system tumors.

### 6.6 Priority Review Voucher (PRV)

While not an exclusivity provision, the priority review voucher (PRV) is nevertheless an incentive to develop new treatments in the specific areas of neglected tropical diseases, rare pediatric diseases, and medical countermeasures only in the United States. Under this program, the Food and Drug Administration awards a priority review voucher to the sponsor of a new drug or vaccine after the FDA approves the product for the prevention or treatment of tropical infectious diseases such as Chagas, malaria, and leishmaniasis, rare pediatric diseases such as spinal muscular atrophy (SMA), and Duchenne muscular dystrophy, and “conditions associated with chemical, biological, radiological, or nuclear (CBRN) threats, emerging infectious diseases, or natural disaster conditions” [54]. The real perceived value of a priority review voucher, which, in theory, shortcuts FDA review times from 10 months to 6 months, is their transferability and salability at exorbitant prices ranging from $67.5-$350 million to other companies for use on different medicines. Since RRx-001 is under development as a medical countermeasure in case of a nuclear or radiological emergency, it may qualify for a PRV if approved for it.

## 7 DATA EXCLUSIVITY IN THE EU

Following the Hatch-Waxman, the EU adopted a regulation in 2004, mandating a period of data exclusivity of at least ten years with the option to extend for another year “if, during the first eight years of those ten years, the (originator) obtains an authorisation for one or more new therapeutic indications which bring a significant clinical benefit in comparison with existing therapies” [44]. Like in the US, the orphan designation is available in the EU. However, the exclusivity period is longer-10 years instead of 7. RRx-001 has been granted an orphan designation in the EU for SCLC.

## 8 DATA EXCLUSIVITY IN JAPAN

Like the US, a patent term extension of up to 5 years is available in Japan for an NCE. However, unlike the US and Europe, it is the patentee, and not the holder of the NDA/market authorization, which must request it but only after a drug approval pertinent to the patent is obtained [55]. No *de jure* exclusivity or data protection system has been established in Japan. However, a re-examination period of approximately 10 years, during which time the benefit/risk balance of the drug is assessed, serves as the *de facto* data exclusivity term [56]. The duration of the re-examination period is 10 years for an orphan drug, 8 years for an NCE, 4 years for new combination drugs or drugs with a new route of administration, and 4-6 years for drugs with a new indication or new dosage. Like the EU, the benefits of orphan exclusivity are 10 years. Regarding pediatric exclusivity none formally exists; however, the re-examination period is extended up to 10 years if clinical trials for children are planned [57]. A comparison of patent and regulatory exclusivities between the US, Europe, and Japan are shown below in Table **1**.

## CONCLUSION

According to Benjamin Franklin in 1789, “In this world, nothing can be said to be certain, except death and taxes” [58]. A contemporary addition to this list is patent and regulatory exclusivities, which are certain, but also temporary. These exclusivities aim to preserve a tenuous balance between innovation and accessibility. On the one hand, exclusivities encourage innovation-and, by extension profit-through restriction on the use of an approved drug. Given how risky it is to develop new biopharmaceutical products, the ability to amortize the expenditures incurred and to reap the full benefits of commercialization is a *sine qua non* condition for corporate survival. However, on the other hand, the expiration of exclusivities opens the door to generic competition; that competition, by usurping brand name hegemony, lowers prices on average by 70-80% and increases accessibility.

An oft-repeated criticism is that these monopolistic exclusivities unfairly hinder competition to the detriment of patients but much to the benefit of pharmaceutical companies, which seem to prioritize profits above all else [59]. However, this criticism goes both ways because without robust incentive mechanisms in place to incentivize pharmaceutical advancement fewer new drugs would be developed for diseases that currently lack effective treatments.

It is the opinion of the authors that drug developers are, by and large, except for a few fraudulent outliers and “bad apples”, like “Pharma Bro”, Martin Shkreli, and Theranos founder, Elizabeth Holmes, caught between the proverbial rock and a hard place, unfairly caricaturized as callous villains and spoiled brats, who are only in it for the money, a sentiment that goes double for the greedy, deceptive pharmaceutical companies which employ them [60]. However, from the authors’ collective experience, this is usually not the case; most in the industry love what they do, empathize with the plight of patients, and intrinsically desire to advance medicine for the betterment of mankind. However, that well-intentioned desire is balanced by a realistic, clear-eyed understanding of the financial resources, which are needed to support the high costs of innovative R&D, including the substantial expense imposed on biopharmaceutical companies by patents and patent litigation fees.

**Fig. (2):**
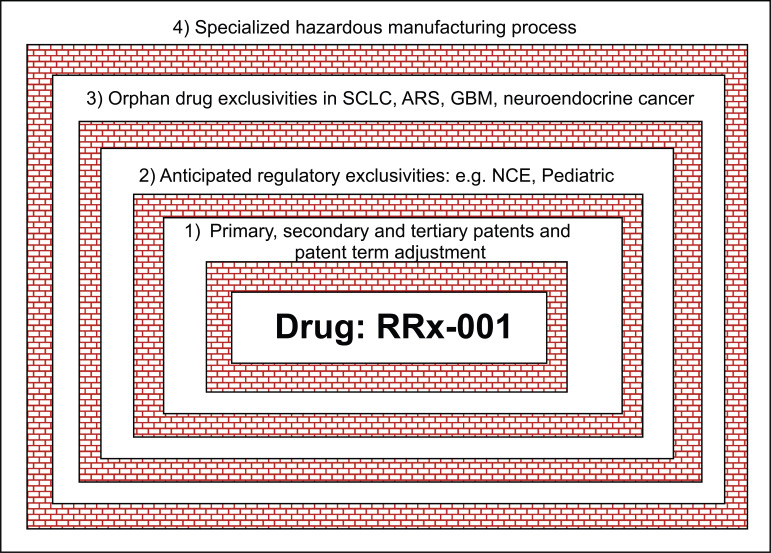
Quadruple Fence Regulatory- and Patent-Protection for RRx-001.

With that in mind, this review was written to educate and inform other drug developers about current regulatory and patent constructs that are available to them in the US, Europe, and Japan to defray costs and to amortize risk. RRx-001, an aerospace-derived NCE, on which the authors have closely collaborated, is used herein as a real-world example. Due to extensive and aggressive use of these provisions, RRx-001 is “triple or quadruple fence” protected, potentially until at least 2042, from 1) primary, secondary and tertiary patents with the right to certain 5-year patent extensions and patent term adjustment 2) an anticipated NCE exclusivity, on approval, of 5 years in the US with an additional 3 years for alternate indications or formulation changes and an extra 0.5 months for pediatric exclusivity and 11 years in Europe 3) orphan indication exclusivity in small cell lung cancer (SCLC), acute radiation protection, neuroendocrine cancer, and glioblastoma (GBM) for 7 years in the US and 10 years (SCLC only) in Europe. A fourth, unofficial barrier to competition is the manufacturing complexity and specialized, new facility investment required for the scaled-up synthesis of RRx-001 to prevent the propagation of explosion or mass detonation (Fig. **2**).

As more data is collected and preclinical research is completed, further method-of-use patent filings and orphan indication submissions are planned not only in the US but also in Europe, and Asia.

Rather than “loopholes”, which are used to “game the system” and to continue the brand name in evergreen perpetuity, as several authors have alleged [61, 62], regulatory, and patent exclusivities are a necessary good (or evil), depending on one’s point of view, which benefits society long-term through more pharmaceutical innovations even if in the short term they temporarily limit patient access to them. The complexity of these issues notwithstanding, the application of regulatory, and patent exclusivities for drug developers are fortunately not, to use a common phrase, “rocket science”. Except that in the specific case of RRx-001, which was sourced from the aerospace industry, it quite literally is rocket science.

## CURRENT AND FUTURE DEVELOPMENTS

That the drug development process is long, expensive, and prone to failure is generally well-known. Also well-known and well-publicized is the overriding demand for new and better therapeutics, which target life-threatening, seriously debilitating, and chronic diseases. Less well-known or understood is how much pharmaceutical companies rely on the quid pro quo of innovation incentives to recoup the considerable investment in the drug development and approval process or to make it in some way worthwhile or attractive for them to go forward. The two most important of these incentives are patents and regulatory exclusions. For RRx-001, which serves as a running example throughout this manuscript, patents are the mainstay mechanism to prevent competition since they grant 20 years of exclusive use. However, the length of the discovery, clinical trial, and approval processes erode the time under which the core “composition of matter” remains in force, even after patent term restoration.

To extend marketing exclusivity beyond the life of its core composition of matter patent, and to prevent misappropriation, companies tend to amass a portfolio of additional patents on polymorphs, formulations, manufacturing processes, dosage forms *etc*.; for RRx-001, in the foreseeable future, patent filings will remain high. As the authors can attest, this strategy is very costly and not necessarily iron-clad from the perspective that, once patents publish, knowledge and dissemination of the invention(s) may inspire other companies or competition to try to invent their way around them, which, however, is difficult to do in the case of RRx-001, given how novel it is and how explosive to manufacture.

What compares favorably with the average length of patent protection post-registration is the period of market exclusivity afforded by regulatory protections. Using RRx-001 as an example, if it is approved in the US, EU, Canada, and Japan for small cell lung cancer or as an anti-mucositis agent in head and neck cancer, it would receive new chemical entity (NCE) status exclusivity for periods of 10 years in the EU, 8 years in Canada, and 5 years in the US and effectively 9 years of post-marketing surveillance protection in Japan. If RRx-001 is registered as a drug for a rare pediatric disease in those markets, such as glioblastoma (GBM), since a clinical trial in GBM with RRx-001 is ongoing, it would also receive concurrent orphan drug exclusivity in the rare disease indication of 7 years in the US, a total of 12 years of orphan drug exclusivity in the EU and an additional 0.5 years of NCE exclusivity in Canada for a total of 8.5 years, and 10 years of orphan drug post-marketing surveillance protection in Japan. Since RRx-001 has also demonstrated antibiotic activity, if it is registered as a new antimicrobial to treat a serious or life-threatening infection, it would gain a 5-year extension of US NCE exclusivity for a total of 10 years of protection. Additionally, RRx-001 will receive additional regulatory exclusivity if it is approved for a second indication outside of small cell lung cancer or head and neck cancer.

Finally, in terms of non-exclusivity-based incentives for drug development, the US awards “priority review vouchers” for a subsequent application that does not itself qualify for priority review on the approval of a treatment for a rare pediatric disease, a neglected tropical disease, for chemical, biological, radiological, and nuclear threats and emerging infectious diseases, which is noteworthy since RRx-001 is under development as a medical countermeasure in case of a radiologic or nuclear emergency. Interestingly, these priority review vouchers may generate as much as USD $350 million in revenue if they are auctioned in the secondary market.

The pharmaceutical industry, to which the authors belong, contends that the primary drivers of innovation in drug development are patent and regulatory exclusivity incentives, without which new promising treatments would likely not appear given the enormous expenditures involved. An urgent question, which looms, is how to strike a balance between the fundamental need—and, daresay right-of pharmaceutical companies to not only turn a profit but also to maintain a profit on the treatments that they develop *versus* how to make these treatments more accessible for the patients that need them.

## Figures and Tables

**Table 1:** Comparison of patent and regulatory exclusivities between the US, Europe, and Japan.

**Patent and Regulatory Exclusivities**	**US**	**EU**	**Japan**
**Basic Patent Term**	20 years	20 years	20 years
**Entitlement to Patent**	First to file (as of 2013)	First to file	First to file
**Patent Term Extension/Supplementary Protection Certificates**	Up to 5 years (with the maximum effective patent life limited to 14 years from product approval)	Up to 5 years plus 6 months for pediatric uses (with the maximum effective patent life limited to 15 years from product approval)	5 years
**NCE Exclusivity**	5 years plus 6 months for pediatric uses; 3 more years for a new clinical indication	11 years	8-10 years
**Pediatric Exclusivity**	Yes (6 months)	Yes (6 months)	No
**Orphan Drug Exclusivity**	7 years	10 years	Up to 10 years
**Priority Review Voucher**	Yes	No	No
**Generic Drug Application Process**	Yes	Yes	No

## LIST OF ABBREVIATIONS

HIV: Human Immunodeficiency Virus
COVID-19: Coronavirus Disease of 2019
NLRP3: NOD-, LRR-, and Pyrin Domain Containing Protein 3
R&D: Research and Development
NCE: New Chemical Entity
NME: New Molecular Entity
NBE: New Biological Entity
EU: European Union
TNAZ: Trinitroazetidine
USAN: United States Adopted Names
INN: International Non-proprietary Names
TRIPS: Trade-Related Aspects of Intellectual Property Rights
USPTO: United States Patent and Trademark Office
EPO: European Patent Office
JPO: Japan Patent Office
KIPO: Korean Intellectual Property Office
PCT: Patent Cooperation Treaty
US: United States
USFDA: United States Federal Drug Administration
ANDA: Abbreviated New Drug Application
sANDA: Supplemental Abbreviated New Drug Application
API: Active Pharmaceutical Ingredient
PT: Patent Term
PTE: Patent Term Extensions
SPC: Supplementary Protection Certificates
PTA: Patent Term Adjustments
ABDNAZ: Alpha Bromo Dinitroazetidine
HMX: Octahydro-1,3,5,7-tetranitro-1,3,5,7-tetrazocine
RDX: Hexahydro-1,3,5-trinitro-1,3,5-triazine
cGMP: Current Good Manufacturing Practices
GRAS: Generally Recognized as Safe
NDA: New Drug Application
sNDA: Supplemental New Drug Application
GAIN: Generating Antibiotic Incentives Now Act
QIDP: Qualified Infectious Disease Products
FD&C: Food, Drug, and Cosmetic
MMA: Medicare Prescription Drug Improvement and Modernization Act
ODA: Orphan Drug Act
ODE: Orphan Drug Exclusivity
PE: Pediatric Exclusivity
OTC: Over The Counter
CIE: Clinical Investigation Exclusivity
GDE: Generic Drug Exclusivity
QIDP: Qualified Infectious Disease Products Exclusivity
PRV: Priority Review Voucher
SMA: Spinal Muscular Atrophy
CBRN: Chemical, Biological, Radiological, Nuclear
ARS: Acute Radiation Syndrome
GBM: Glioblastoma
SCLC: Small Cell Lung Cancer
